# A systematic review of quality of reporting in registered intimate partner violence studies: where can we improve?

**DOI:** 10.5249/jivr.v11i2.1140

**Published:** 2019-07

**Authors:** Kim Madden, Mark Phillips, Max Solow, Victoria McKinnon, Mohit Bhandari

**Affiliations:** ^*a*^Department of Health Research Methods, Evidence, and Impact, McMaster University, Hamilton, Ontario, Canada.; ^*b*^Centre for Evidence-Based Orthopedics, Department of Surgery, McMaster University, Hamilton, Ontario, Canada.; ^*c*^School of Medicine, St. George’s University, Grenada.; ^*d*^Department of Medicine, McMaster University, Hamilton, Ontario, Canada.

**Keywords:** Intimate partner violence, Spouse abuse, Transparency, Randomized controlled trials, Pilot studies

## Abstract

**Background:**

Reporting quality is paramount when presenting clinical findings in published research to ensure that we have the highest quality of evidence. Poorly reported clinical findings can result in a number of potential pitfalls, including confusion of the methodology used or selective reporting of study results. There are guidelines and checklists that aim to standardize the way in which studies are reported in the literature to ensure transparency. The use of these reporting guidelines may aid in the appropriate reporting of research, which is of increased importance in highly complex fields like intimate partner violence (IPV). The primary objective of this systematic review is to assess the reporting quality of published IPV studies using the CONSORT and STROBE checklists.

**Methods:**

We performed a systematic review of three large study registries for IPV studies. Of the completed studies, we sought full text publications and used reporting checklists to assess the quality of reporting.

**Results:**

Of the 42 randomized controlled trials, the mean score on the CONSORT checklist was 63.5% (23.5/37 items, SD 4.7 items). There were also 12 pilot trials in this systematic review, which scored a mean of 49.3% (19.7/40 items; SD 3.3 items) on the CONSORT extension for pilot trials. We included 12 observational studies which scored a mean of 56.1% (18.5/33 items; SD: 4.1 items).

**Conclusions:**

We identified an opportunity to improve reporting quality by encouraging adherence to reporting guidelines. There should be a particular focus on ensuring that pilot studies report pilot-specific items. All researchers have a responsibility to ensure commitment to high quality reporting to ensure transparency in IPV studies.

## Introduction

Intimate partner violence (IPV) refers to behavior by an intimate partner or ex-partner that causes physical, sexual or psychological harm, including physical aggression, sexual coercion, psychological abuse, and controlling behaviors.^1^ IPV is a human rights violation that affects men and women of all walks of life and is pervasive worldwide. More than one third of female homicides globally are perpetrated by an intimate partner,^2^ and IPV is a prevalent source of non-fatal injury to women.^3^ To address the need for health care professionals to assist victims of abuse, multiple IPV screening, identification, advocacy, and assistance programs have been developed and implemented across different clinical settings. A variety of research methodologies and outcome measures have been used to evaluate each program’s effectiveness. The results of these studies are often inconclusive and frequently conflicting, resulting in a high level of clinical uncertainty and controversy regarding the merits of IPV screening and assistance programs.^4-6^ Because of the clinical importance of IPV, controversies in the field, and the need for high quality evidence to resolve these controversies, it is important to focus on the quality of research including reporting quality.

Quality of reporting is paramount when presenting clinical findings in published research to ensure that we have the highest quality of evidence on this important topic. Poorly reported clinical findings can result in a number of potential pitfalls, including confusion of the methodology used or selective reporting of study results.^7,8^ High quality reporting is a key aspect of research transparency. Studies that are inadequately reported may also score poorly on risk of bias assessments due to lack of clarity in the published manuscript.^9^ The Consolidated Standards of Reporting (CONSORT) checklist is a tool that aims to standardize the way in which randomized trials are reported in the literature to ensure transparency.^7^ Other checklists for other study designs have also been developed for the same purpose, including Strengthening Reporting of Observational Studies in Epidemiology (STROBE) for observational studies,^10,11^ Preferred Reporting Items for Systematic Reviews and Meta-Analyses (PRISMA) for systematic reviews,^12,13^ and others. The use of these reporting guidelines and checklists may aid in the appropriate reporting of research, which is of increased importance in fields that have controversies and complex methodological issues, such as intimate partner violence.

The primary objective of this systematic review is to assess the reporting quality of published IPV studies. Our overarching goal is to determine which aspects of reporting are commonly deficient so that we can make recommendations to improve the transparency and clarity of IPV research in the future. 

## Methods 

This is a secondary analysis of a previously published systematic review^14,15^ which answers a different research question than the original review. The methods are described below. 

**Study Inclusion**

We performed a search of the three largest English-language registries, clinicaltrials.gov, the Netherlands Trial Registry (NTR), and Current Controlled Trials (ISRCTN) on September 12, 2017 using the terms "spouse abuse" OR "domestic violence" OR "partner violence" OR "partner abuse". Two reviewers independently reviewed all identified registry records for possibly eligible studies. We included registry records for studies of any design for which the date of completion was at least 18 months prior to the search date. We chose a cut-off of 18 months to allow sufficient lag time between reporting the study is complete and publication. We excluded registry records if they focused only on child abuse, or if the title, outcomes, interventions, and conditions did not mention intimate partner violence or a related term such as domestic violence. We had no date restrictions, although it was uncommon to register non-drug trials before 2006. Non-interventional studies are not required to be registered; however, investigators are permitted to register them for transparency. We chose to include non-interventional study records in this review for completeness.

**Identification of Publications**

Two authors independently attempted to locate each publication to match the included trial records. We searched AMED (Allied and Complementary Medicine Database), Embase, Global Health, Healthstar, Medline, and PsycInfo using the Ovid search interface, plus Google Scholar for the matching publications. We also attempted on up to three occasions to contact the Principal Investigator listed on the trial registry record for publications that could not be located and publications for which it was unclear if they matched the registry record. We included all published studies as long as they reported a primary outcome (i.e., not just feasibility or baseline characteristics), including preliminary findings. In case of disagreement between the two reviewers, a senior author broke the tie.

**Assessment of Reporting Completeness**

Two authors independently completed the CONSORT checklist for randomized controlled trials (RCTs), or the STROBE checklist for observational studies, and conflicts were resolved through discussion or consulting a more senior reviewer. The CONSORT checklist includes 37 items addressing completeness of reporting of the title/abstract, background/objectives, design, participants, interventions, outcomes, randomization and blinding considerations, sample size and statistical considerations, recruitment and retention, and discussion items. For pilot RCTs, we used the CONSORT extension for pilot and feasibility studies which has language that is adapted for pilot studies including feasibility objectives/outcomes, feasibility success criteria, and rationale for why a pilot trial is needed.^16,17^ The STROBE checklist is a 33 item list that is similar to CONSORT but tailored for observational studies. For example, randomization and blinding do not apply to observational studies so those items are removed, there is more emphasis on controlling confounding, and the wording is tailored to the three major types of observational studies: cohort studies, case-control studies, and cross-sectional studies. We awarded 1 point for complete reporting of the item, 0.5 points for reporting with weaknesses, and 0 points for items that were not reported. In case of disagreement between the two reviewers, a senior author broke the tie.

**Data Analysis**

The analyses are descriptive. We present frequency data (proportions and percentages) to describe the percentage of studies that fully reported, partially reported, and did not report each checklist item. We also report the mean and standard deviation of reported items for each study. 

## Results

**Literature Search Results**

**Figure 1:**
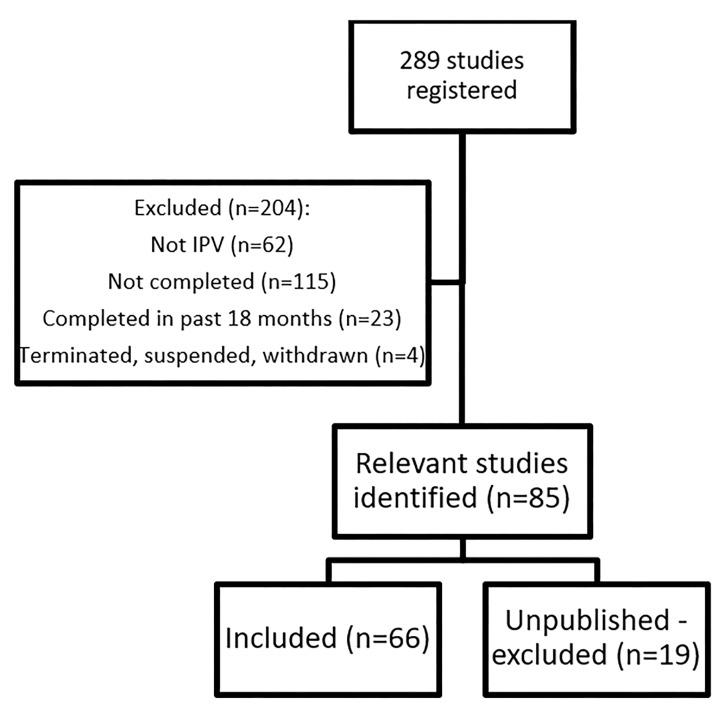
Study flow diagram.

Our search of clinicaltrials.gov and ISRCTN revealed 289 possibly eligible studies. We found no relevant studies in NTR. 204 of these studies were ineligible because they were unrelated to IPV or they were still ongoing. We excluded 19 registered studies because they had no associated published paper. We included a total of 66 studies from clinicaltrials.gov and ISRCTN (Figure 1;Appendix 1). 42 studies (63.6%) were definitive randomized trials, 12 (18.2%) were pilot/feasibility trials, and 12 (18.2%) were observational studies. Of the 42 definitive randomized trials, 20 (47.6%) were 2 group parallel trials, 5 (11.9%) were 3 or 4 group parallel trials, 12 (28.6%) were cluster randomized trials, 1 (2.4%) was a parallel trial embedded in a mixed methods study, and 4 (9.5%) were unclear in their study design.

**Reporting Completeness – Definitive Trials**

For the 42 definitive randomized controlled trials, the mean number of correctly reported items was 23.5 (SD: 4.7; 95% CI: 22.0 to 25.0) out of 37 items (63.5%). The only item that was reported fully in each study was the scientific background. Other items that were generally well-reported included interventions, interpretation consistent with results, settings and locations, numbers randomized and receiving interventions, and limitations. The lowest scoring items in terms of reporting were changes in methods, changes in outcomes, harms, and where the protocol can be accessed (Table 1).

**Table 1:** Quality of Reporting for Definitive Randomized Trials (CONSORT).

CONSORT Item n=42 trials	Fully Reported n (%)	Partially Reported n (%)	Not Reported n (%)
Identified as randomized trial in title	30 (71.4)	2 (4.8)	10 (23.8)
Structured abstract	36 (85.7)	6 (14.3)	0 (0)
Scientific background and rationale	42 (100)	0 (0)	0 (0)
Specific objectives	37 (88.1)	1 (2.4)	4 (9.5)
Description of design	17 (40.5)	14 (33.3)	11 (26.2)
Changes to methods	4 (9.5)	0 (0)	38 (90.5)
Eligibility criteria	38 (90.5)	1 (2.4)	3 (7.1)
Settings and locations	38 (90.5)	3 (7.1)	1 (2.4)
Intervention description	40 (95.2)	1 (2.4)	1 (2.4)
Primary and secondary outcomes	38 (90.5)	1 (2.4)	3 (7.1)
Changes to outcomes or measurements	0 (0)	0 (0)	42 (100)
Rationale for sample size	19 (45.2)	2 (4.8)	21 (50.0)
Interim analysis and stopping guidelines	2 (4.8)	1 (2.4)	39 (92.9)
Methods to generate randomization sequence	22 (52.4)	1 (2.4)	19 (45.2)
Type of randomization	18 (42.9)	1 (2.4)	23 (54.8)
Mechanism to implement randomization	17 (40.5)	1 (2.4)	24 (57.1)
Who was responsible for randomization/enrollment steps	13 (31.0)	1 (2.4)	28 (66.7)
Who was blinded	12 (28.6)	4 (9.5)	26 (61.9)
Similarity of interventions	5 (11.9)	0 (0)	37 (88.1)
Statistical methods for primary and secondary outcomes	39 (92.9)	1 (2.4)	2 (4.8)
Additional analysis methods (subgroups, adjusted etc.)	30 (71.4)	0 (0)	12 (28.6)
Participant flow	39 (92.9)	1 (2.4)	22 (52.4)
Losses and exclusions	33 (78.6)	4 (9.5)	5 (11.9)
Recruitment and follow-up dates	35 (83.3)	1 (2.4)	6 (14.3)
Why trial stopped	5 (11.9)	3 (7.1)	34 (81.0)
Baseline demographics	37 (88.1)	1 (2.4)	4 (9.5)
Denominator for each outcome	30 (71.4)	4 (9.5)	8 (19.0)
Results and uncertainty (e.g. 95% CI) for each outcome	34 (81.0)	8 (19.0)	0 (0)
Present absolute and relative risks	8 (19.0)	2 (4.8)	32 (76.2)
Results of other analyses (subgroups, adjusted etc.)	36 (85.7)	0 (0)	6 (14.3)
Harms	5 (11.9)	2 (4.8)	35 (83.3)
Limitations	38 (90.5)	2 (4.8)	2 (4.8)
Generalizability	36 (85.7)	4 (9.5)	2 (4.8)
Interpretation consistent with results	41 (97.6)	1 (2.4)	0 (0)
Registration number	31 (73.8)	0 (0)	11 (26.2)
Where protocol can be accessed	5 (11.9)	0 (0)	37 (88.1)
Funders	38 (90.5)	0 (0)	4 (9.5)

**Reporting Completeness – Pilot/Feasibility Trials**

For the 12 pilot trials, the mean number of correctly reported items was 19.7 (SD: 3.3; 95% CI: 17.6 to 23.8) of 40 (49.3%). Two items were reported fully in each study: settings/locations, and interventions for each group. Other items that were generally well-reported included identifying the study as a pilot in the title and reporting limitations. The lowest scoring items were description of pilot design including allocation ratio, methodological changes after trial commencement, criteria to judge to proceed to definitive trial, rationale for sample size, interim analyses and stopping guidelines, blinding, why the trials was stopped, harms, registration number, and where the protocol can be accessed (Table 2).

**Table 2:** Quality of Reporting for Pilot Randomized Trials (CONSORT Pilot).

CONSORT Item – Pilot extension n=12 pilot trials	Fully Reported n (%)	Partially Reported n (%)	Not Reported n (%)
Identified as pilot trial in title	10 (83.3)	2 (16.7)	0 (0)
Structured abstract	3 (25.0)	9 (75.0)	0 (0)
Scientific background and rationale for pilot	0 (0)	12 (100)	0 (0)
Specific objectives for pilot	4 (33.3)	5 (41.7)	3 (25.0)
Description of pilot design	5 (41.7)	0 (0)	7 (58.3)
Changes to methods	1 (8.3)	0 (0)	11 (91.7)
Eligibility criteria	10 (83.3)	0 (0)	2 (16.7)
Settings and locations	12 (100)	0 (0)	0 (0)
How participants identified and consented	10 (83.3)	0 (0)	2 (16.7)
Intervention description	12 (100)	0 (0)	0 (0)
Measurement of all outcomes	3 (25.0)	9 (75.0)	0 (0)
Changes to outcomes or measurements	0 (0)	0 (0)	12 (100)
Criteria for whether/how to proceed to definitive trial	0 (0)	0 (0)	12 (100)
Rationale for sample size	0 (0)	0 (0)	12 (100)
Interim analysis and stopping guidelines	0 (0)	0 (0)	12 (100)
Methods to generate randomization sequence	5 (41.7)	0 (0)	7 (58.3)
Type of randomization	3 (25.0)	1 (8.3)	8 (66.7)
Mechanism to implement randomization	4 (33.3)	1 (8.3)	7 (58.3)
Who was responsible for randomization/enrollment steps	1 (8.3)	3 (25.0)	8 (66.7)
Who was blinded	1 (8.3)	0 (0)	11 (91.2)
Similarity of interventions	0 (0)	0 (0)	12 (100)
Statistical methods	9 (75.0)	3 (25.0)	0 (0)
Participant flow	10 (83.3)	1 (8.3)	1 (8.3)
Losses and exclusions	10 (83.3)	1 (8.3)	1 (8.3)
Recruitment and follow-up dates	6 (50.0)	1 (8.3)	5 (41.7)
Why trial stopped	0 (0)	0 (0)	12 (100)
Baseline demographics	9 (75.0)	1 (8.3)	2 (16.7)
Denominator for each outcome	11 (91.2)	0 (0)	1 (8.3)
Results and uncertainty (e.g. 95% CI) for each outcome	6 (50.0)	6 (50.0)	0 (0)
Results of other analyses	8 (66.7)	1 (8.3)	3 (25.0)
Harms	2 (16.7)	0 (0)	10 (83.3)
Unintended consequences	1 (8.3)	0 (0)	11 (91.2)
Limitations and feasibility uncertainty	11 (91.2)	0 (0)	1 (8.3)
Generalizability	9 (75.0)	0 (0)	3 (25.0)
Interpretation consistent with results	10 (83.3)	2 (16.7)	0 (0)
Progression to definitive	4 (33.3)	1 (8.3)	7 (58.3)
Registration number	2 (16.7)	0 (0)	10 (83.3)
Where protocol can be accessed	1 (8.3)	0 (0)	11 (91.2)
Funders and role	0 (0)	11 (91.2)	1 (8.3)
Ethical approval	8 (66.7)	1 (8.3)	3 (25.0)

**Reporting Completeness – Observational Studies**

For the 12 observational studies, the mean number of correctly reported items was 18.5 (SD: 4.1; 95% CI: 15.9 to 21.1) of 33 (56.1%). The only item that was reported fully in each study was numbers of outcome and exposure events. Other items that were generally well-reported included summarizing the results in the discussion, discussing the limitations of the study, explaining the scientific background and rationale, and describing the statistical methods. The lowest scoring items in terms of reporting were indicating the design in the title, explaining how loss to follow-up was addressed, and reporting both relative and absolute risks(Table 3).

**Table 3:** Quality of Reporting for Observational Studies (STROBE).

STROBE Item n=12 observational studies	Fully Reported n (%)	Partially Reported n (%)	Not Reported n (%)	Not applicable n (%)
Study design in title	3 (25.0)	0 (0)	9 (75.0)	
Informative and balanced abstract	7 (58.3)	5 (41.7)	0 (0)	
Scientific background and rationale	9 (75.0)	3 (25.0)	0 (0)	
Specific objectives	8 (66.7)	4 (33.3)	0 (0)	
Key elements of study design early in paper	8 (66.7)	2 (16.7)	2 (16.7)	
Setting, locations, dates	7 (58.3)	4 (33.3)	1 (8.3)	
Eligibility criteria	7 (58.3)	2 (16.7)	3 (25.0)	
Define outcomes, exposures, predictors, confounders	7 (58.3)	5 (41.7)	0 (0)	
Sources of data an measurement methods	7 (58.3)	5 (41.7)	0 (0)	
Describe efforts to address bias	6 (50.0)	1 (8.3)	5 (41.7)	
Explain sample size	2 (16.7)	1 (8.3)	9 (75.0)	
How quantitative variables were handled	7 (58.3)	5 (41.7)	0 (0)	
Statistical methods	9 (75.0)	3 (25.0)	0 (0)	
Methods for subgroups and interactions	5 (41.7)	0 (0)	7 (58.3)	
How missing data addressed	0 (0)	1 (8.3)	11 (91.7)	
How loss to follow-up addressed	0 (0)	0 (0)	6 (50.0)	6 (50.0)
Sensitivity analysis methods	0 (0)	0 (0)	12 (100)	
Numbers of participants at each stage	5 (41.7)	7 (58.3)	0 (0)	
Reasons for non-participation	3 (25.0)	2 (16.7)	7 (58.3)	
Flow diagram	2 (16.7)	1 (8.3)	9 (75.0)	
Participant characteristics	10	0 (0)	2 (16.7)	
Numbers of participants with missing data	1 (8.3)	1 (8.3)	10 (83.3)	
Summarize follow-up time	6 (50.0)	0 (0)	0 (0)	6 (50.0)
Report numbers of outcome/exposure events	12 (100)	0 (0)	0 (0)	
Unadjusted estimates and precision	8 (66.7)	2 (16.7)	2 (16.7)	
Category boundaries for continuous variables that were categorized	5 (41.7)	0 (0)	0 (0)	7 (58.3)
Relative risk and absolute risk	0 (0)	0 (0)	12 (100.0)	
Other analyses	8 (66.7)	1 (8.3)	3 (25.0)	
Summarize key results	11 (91.7)	1 (8.3)	0 (0)	
Limitations	11 (91.7)	0 (0)	1 (8.3)	
Cautious overall interpretation	8 (66.7)	3 (25.0)	1 (8.3)	
Generalizability	3 (25.0)	7 (58.3)	2 (16.7)	
Source of funding and role of funders	3 (25.0)	4 (33.3)	5 (41.7)	

## Discussion

In this systematic review of 66 IPV studies, we found that reporting guidelines were followed well in some cases but not very well in other cases. Of the 42 randomized controlled trials, the mean score on the CONSORT checklist was 63.5% (23.5/37 items, SD 4.7 items). There were also 12 pilot trials in this systematic review, which scored a mean of 49.3% (19.7/40 items; SD 3.3 items) on the CONSORT extension for pilot trials. We included 12 observational studies which scored a mean of 56.1% (18.5/33 items; SD: 4.1 items). In each of the three study types, limitations were well-explained. In interventional studies, the settings/locations, and interventions for each group were well-described in most trials. The scientific background was also done well in definitive trials and observational studies. However, this section did not score highly in pilot trials because the pilot extension also requires an explanation for why a pilot is needed, and that was generally not well-reported. The items that were generally poorly reported were changes that occurred after study commencement, where the protocol can be accessed, and harms of interventions for interventional studies. In addition, the pilot-specific items were generally not well-reported, including rationale for a pilot design, criteria for feasibility success, and feasibility objectives.

There have been numerous previous studies that have assessed adherence to the CONSORT statement and checklist, including acupuncture,^18^ prosthodontics,^19^ nursing,^20^ cardiology,^21^ and many others. These studies consistently demonstrate suboptimal reporting in nearly every field, but we are unaware of any similar studies in the IPV field. There have also been studies of adherence to STROBE, including general medicine,^22^ occupational medicine,^23^ influenza^24^ and others which show a similar trend of suboptimal reporting. There have not been many studies to date assessing the quality of pilot trial reporting using the CONSORT pilot extension. However, a study of pilot cluster RCTs showed similar results to the current study, particularly that there is a lack of emphasis on feasibility-specific items.^25^ Additionally, previous studies focusing on harms of interventions have found similar results, particularly that harms are poorly reported in published trials.^26,27^

These findings that study reporting is generally poor, which is consistent across specialties and study designs, suggests that further emphasis needs to be placed on adherence to reporting guidelines. Even though many journals and the International Committee of Medical Journal Editors (ICMJE) endorse reporting guidelines, authors still do not adhere to the guidelines. Poor reporting is still an issue even when authors are required to complete and submit a CONSORT checklist (or other checklist depending on study design) with their manuscript.^28^ It has been suggested that editorial assistants should be responsible to ensuring compliance with reporting guidelines^28^ and we suggest that peer reviewers should be trained to ensure that all items are reported. Another study showed that CONSORT adherence was improved when a dental journal required the use of specific subheadings that follow CONSORT requirements.^26^ This could be implemented in other specialties to enhance reporting quality, but would require individual journals to agree to the change, and it would require subheadings to be tailored for other study designs. 

These findings that IPV studies are not well-reported are not a purely editorial issue. Studies that are not well-reported are vulnerable to misunderstanding, bias, and conflicts of interest among other things. If it is difficult to interpret or understand the IPV literature because of poor reporting, clinicians will be unable to use the information in their practice or the information will be misleading. If harms of an intervention are not reported properly in a study, the intervention may be adopted into clinical practice without critical information about possible drawbacks. If there are unreported conflicts of interest, such as industry influence, clinicians could adopt an intervention into practice without knowledge of the industry bias and the ramifications thereof. Additionally, poor reporting makes it difficult for systematic review and clinical practice guideline authors to make appropriate decisions regarding the available literature. It is possible that otherwise good studies could be discarded due to poor reporting, and will fail to make an impact in the field. All of these drawbacks of poor reporting make it more difficult for clinicians to implement evidence-based interventions or programs, which can negatively affect the victims of IPV in two ways: failure to implement a high-quality intervention/program; or implementing a harmful or ineffective intervention/program.

Although we followed a systematic process to complete this review, with duplicate reviewers and attempts to limit errors, there are some limitations. We focused only on studies that were registered in clinicaltrials.gov or ISRCTN and were subsequently published. Studies that were not registered, particularly non-randomized studies, were likely left out and may be different than included studies in important ways. Additionally, some items are subjective to rate; particularly the ones that could be judged "partially reported". We attempted to limit this effect by requiring data extractors to train with the lead author prior to completing data extraction assignments, and having two independent assessors. 

## Conclusion

In this systematic review of IPV studies we identified that there is an opportunity to improve reporting quality and transparency by encouraging adherence to reporting guidelines such as CONSORT and STROBE. Additionally, there should be a particular focus on ensuring that pilot studies report pilot-specific items, specifically rationale for a pilot design, criteria for feasibility success, and feasibility objectives. Journal editing staff, peer reviewers, and authors all have a responsibility to ensure commitment to high quality reporting to ensure transparency in IPV studies.

**Acknowledgements**

We would like to thank Ms. Jane Fox for her assistance with data extraction and Ms. Kerry Tai for her assistance with the literature search.

**Appendix 1.** Published Studies Included in Analyses

Reference
Abramsky T, Devries KM, Michau L, Nakuti J, Musuya T, Kyegombe N, Watts C. The impact of SASA!, a community mobilization intervention, on women’s experiences of intimate partner violence: secondary findings from a cluster randomised trial in Kampala, Uganda. J Epidemiol Community Health 2016;70:818–825.
Ahamd F, Hogg-Johnson S, Stewart DE, Skinner HA, Glazier RH, Levinson W. Computer-Assisted Screening for Intimate Partner Violence and Control: A Randomized Trial. Ann Intern Med. 2009;151:93-102.
Aupperle RL, Allard CB, Simmons AN, Flagan T, Thorp SR, Norman SB, Paulus MP, Stein MB. Neural responses during emotional processing before and after cognitive trauma therapy for battered women. Psychiatry Res. 2013 Oct 30;214(1):48-55.
Bair-Merritt MH, Feudtner C, Mollen CJ, Winters S, Blackstone M, Fein JA. Screening for intimate partner violence using an audiotape questionnaire: a randomized clinical trial in a pediatric emergency department. Arch Pediatr Adolesc Med. 2006 Mar;160(3):311-6.
Bass JK, Annan J, McIvor Murray S, Kaysen D, Griffiths S, Cetinoglu T, Wachter K, Murray LK, Bolton PA. Controlled Trial of Psychotherapy for Congolese Survivors of Sexual Violence. N Engl J Med 2013;368:2182-91.
Becker S, Mlay R, Schwandt HM, Lyamuya E. Comparing couples' and individual voluntary counseling and testing for HIV at antenatal clinics in Tanzania: a randomized trial. AIDS Behav. 2010 Jun;14(3):558-66.
Braithwaite SR, Fincham FD. Computer-based prevention of intimate partner violence in marriage. Behav Res Ther. 2014 Mar;54:12-21.
Brothers J, Hotton AL, Hosek SG, Harper GW, Fernandez I. Young Women Living with HIV: Outcomes from a Targeted Secondary Prevention Empowerment Pilot Trial. AIDS Pat Care STD. 2016; 30(5), 229-235.
Buller AM, Hidrobo M, Peterman A, Heise L. The way to a man’s heart is through his stomach?: a mixed methods study on causal mechanisms through which cash and in-kind food transfers decreased intimate partner violence. BMC Public Health (2016) 16:488.
Calderón SH, Gilbert P, Jackson R, Kohn MA, Gerbert B. Cueing prenatal providers effects on discussions of intimate partner violence. Am J Prev Med. 2008 Feb;34(2):134-7.
Carter PM, Walton MA, Zimmerman MA, Chermack ST, Roche JS, Cunningham RM. Efficacy of a Universal Brief Intervention for Violence Among Urban Emergency Department Youth. Acad Emerg Med 2016 23(9) 1061-1070.
Choo EK, Zlotnick C, Strong DR, Squires DD, Tape C, Mello MJ. BSAFER: A Web-based intervention for drug use and intimate partner violence demonstrates feasibility and acceptability among women in the emergency department. Substance Abuse, 37:3, 441-449.
Coker AL, Flerx VC, Smith PH, Whitaker DJ, Fadden MK, Williams M. Partner violence screening in rural health care clinics. Am J Public Health. 2007 Jul;97(7):1319-25.
Creinin MD, Schreiber CA, Bedmarek P, Lintu H, Wagner MS, Meyn LA. Mifepristone and Misoprostol Administered Simultaneously Versus 24 Hours Apart for Abortion: A Randomized Controlled Trial. Obstet Gynecol 2007 109(4) 885-894.
Doherty IA, Myers B, Zule WA, Minnis AM, Kline TL, Parry CD, El-Bassel N, Weschberg WM. Seek, Test and Disclose: knowledge of HIV testing and serostatus among high-risk couples in a South African township. Sex Transm Infect 2016;92: 5–11.
Feder G, Davies RA, Baird K, Dunne D, Eldridge S, Griffiths C, Gregory A, Howell A, Johnson M, Ramsay J, Rutterford C, Sharp D. Identification and Referral to Improve Safety (IRIS) of women experiencing domestic violence with a primary care training and support programme: a cluster randomised controlled trial. Lancet. 2011 Nov 19;378(9805):1788-95.
George DT, Phillips MJ, Lifshitz M, Lionetti TA, Spero DE, Ghassemzedeh N, Doty L, Umhau JC, Rawlings RR. Fluoxetine treatment of alcoholic perpetrators of domestic violence: a 12-week, double-blind, randomized, placebo-controlled intervention study. J Clin Psychiatry. 2011 Jan;72(1):60-5.
Gilbert L, Shaw SA, Goddard-Eckrich D, Chang M, Rowe J, McCrimmon T, Almonte M, Goodwin S, Epperson M. Project WINGS (Women Initiating New Goals of Safety): A randomized controlled trial of a screening, brief intervention and referral to treatment (SBIRT) service to identify and address intimate partner violence victimisation among substance-using women receiving community supervision. Crim Behav Ment Health 2015 25: 314–329.
Gillum TL, Sun CJ, Woods AB. Can a health clinic-based intervention increase safety in abused women? Results from a pilot study. J Womens Health (Larchmt). 2009 Aug;18(8):1259-64.
Gupta J, Falb KL, Lehmann H, Kpebo D, Xuan Z, Hossain M, Zimmerman C, Watts C, Annan J. Gender norms and economic empowerment intervention to reduce intimate partner violence against women in rural Côte d'Ivoire: a randomized controlled pilot study. BMC Int Health Hum Rights. 2013 Nov 1;13:46.
Gupta J, Falb KL, Ponta O, Xuan Z, Abril Campos P, Arellano Gomez A, Valades J, Cariño G, Diaz Olavarrieta C. A nurse-delivered, clinic-based intervention to address intimate partner violence among low-income women in Mexico City: findings from a cluster randomized controlled trial. BMC Medicine (2017) 15:128.
Haberland N, Ndwiga C, McCarthy K, Makanyengo M, Kosgei R, Choi C, Pulerwitz J, Kalibala S. Addressing intimate partner violence and power in relationships in HIV testing services: Results of an intervention piloted in Nairobi, Kenya. 2016 HIVCore Final Report. Washington, DC: USAID | Project Search: HIVCore.
Hossain M, Zimmerman C, Kiss L, Abramsky T, Kone D, Bakayoko-Topolska M, Annan J, Lehmann H, Watts C. Working with men to prevent intimate partner violence in a conflict-affected setting: a pilot cluster randomized controlled trial in rural Côte d'Ivoire. BMC Public Health. 2014 Apr 10;14:339.
Houry D, Kemball R, Rhodes KV, Kaslow NJ. Intimate partner violence and mental health symptoms in African American female ED patients. Am J Emerg Med. 2006 Jul;24(4):444-50.
Houry D, Kaslow NJ, Kemball RS, McNutt LA, Cerulli C, Straus H, Rosenberg E, Lu C, Rhodes KV. Does screening in the emergency department hurt or help victims of intimate partner violence? Ann Emerg Med. 2008 Apr;51(4):433-42,442.e1-7.
Jaindl M, Endler G, Marculescu R, Eder S, Heisinger S, Kovar FM. Intimate Partner Violence and a New Screening Score – A Prospective Observation Study Over Eight Years. SAS J. Surg., Volume-2; Issue-6 (Nov-Dec, 2016);p-278-286.
Jewkes R, Nduna M, Levin J, Jama N, Dunkle K, Puren A, Duvvury N. Impact of stepping stones on incidence of HIV and HSV-2 and sexual behavior in rural South Africa: cluster randomised controlled trial. BMJ. 2008 Aug 7;337:a506.
Johnson DM, Zlotnick C, Perez S. Cognitive behavioral treatment of PTSD in residents of battered women's shelters: results of a randomized clinical trial. J Consult Clin Psychol. 2011 Aug;79(4):542-51.
Kiely M, El-Mohandes AAE, El-Khorazaty MN, Gantz MG. An Integrated Intervention to Reduce Intimate Partner Violence in Pregnancy: A Randomized Controlled Trial. Obstet Gynecol 2010;115:273–83).
Klevens J, Kee R, Trick W, Garcia D, Angulo FR, Jones R, Sadowski LS. Effect of screening for partner violence on women's quality of life: a randomized controlled trial. JAMA. 2012 Aug 15;308(7):681-9.
Kornfeld BD, Bair-Merritt MH, Frosch E, Solomon BS. Postpartum depression and intimate partner violence in urban mothers: co-occurrence and child healthcare utilization. J Pediatr. 2012 Aug;161(2):348-53.e2.
Kraanen FL, Vedel E, Scholing A, Emmelkamp PMG. The comparative effectiveness of Integrated treatment for Substance abuse and Partner violence (I-StoP) and substance abuse treatment alone: a randomized controlled trial. BMC Psychiatry 2013,13:189.
Levesque DA, Johnson JL, Welch CA, Prochaska JM, Paiva AL. Teen Dating Violence Prevention: Cluster-Randomized Trial of Teen Choices, an Online, Stage-Based Program for Healthy, Nonviolent Relationships. Psychol Violence. 2016 July;6(3):421–432.
MacMillan HL, Wathen CN, Jamieson E, Boyle MH, Shannon HS, Ford-Gilboe M, Worster A, Lent B, Coben JH, Campbell JC, McNutt LA, McMaster Violence Against Women Research Group. Screening for Intimate Partner Violence in Health Care Settings: A Randomized Trial. JAMA. 2009;302(5):493-501.
MacMillan HL, Wathen CN, Jamieson E, Boyle M, McNutt LA, Worster A, Lent B, Webb M; McMaster Violence Against Women Research Group. Approaches to screening for intimate partner violence in health care settings: a randomized trial. JAMA. 2006 Aug 2;296(5):530-6.
Meffert SM, Abdo AO, Alla OAA, Elmakki YOM, Metzler TJ, Marmar CR. A Pilot Randomized Controlled Trial of Interpersonal Psychotherapy for Sudanese Refugees in Cairo, Egypt. Psych Trauma: Res Pract Policy 2014;6(3)240–9.
Miller E, Goldstein S, McCauley HL, Jones KA, Dick RN, Jetton J, Silverman JG, Blackburn S, Monasterio E, James L, Tancredi DJ. A School Health Center Intervention for Abusive Adolescent Relationships: A Cluster RCT. Pediatrics 2015,135(1),76-85.
Miller E. Tancredi DJ, Decker MR, McCauley HL, Jones KA, Anderson H, James L, Silverman JG. A family planning clinic-based intervention to address reproductive coercion: a cluster randomized controlled trial. Contraception 94 (2016) 58–67.
Miller E, Tancredi DJ, McCauley HL, Decker MR, Virata MC, Anderson HA, Stetkevich N, Brown EW, Moideen F, Silverman JG. "Coaching boys into men": a cluster-randomized controlled trial of a dating violence prevention program. J Adolesc Health. 2012 Nov;51(5):431-8.
Mittal M, Thevenet-Morrison K, Landau J, Cai X, Gibson L, Schroeder A, Chaize J, Carey MP. An Integrated HIV Risk Reduction Intervention for Women with a History of Intimate Partner Violence: Pilot Test Results. AIDS Behav (2017) 21:2219–2232.
Murphy CM, Eckhardt CI, Clifford JM, Lamotte AD, Meis LA. Individual Versus Group Cognitive-Behavioral Therapy for Partner- Violent Men: A Preliminary Randomized Trial. 2017 [epub].
Muzny CA, Austin EL, Harbison HS, Hook EW. Sexual Partnership Characteristics of African American Women Who Have Sex With Women; Impact on Sexually Transmitted Infection Risk. Sexually Transmitted Diseases 2014,41(10)611-617.
Myers US Browne KC, Norman SB. Treatment Engagement: Female Survivors of Intimate Partner Violence in Treatment for PTSD and Alcohol Use Disorder. J Dual Diag, 11(3-4), 238–247, 2015.
Padala PR, Madison J, Monnahan M, Marcil W, Price P, Ramaswamy S, Din AU, Wilson DR, Petty F. Risperidone monotherapy for post-traumatic stress disorder related to sexual assault and domestic abuse in women. Int Clin Psychopharmacol. 2006 Sep;21(5):275-80.
Post LA, Raile ANW, Zeoli AM, Taylor R, Smith PK, Dziura JD, Biroscak BJ. Domestic Violence Homicide: Validating a Scale to Measure Implicit Collusion with Murder. Health Sci Res. 2015; 2(1): 1-8.
Pronyk PM, Hargreaves JR, Kim JC, Morison LA, Phetla G, Watts C, Busza J, Porter JD. Effect of a structural intervention for the prevention of intimate-partner violence and HIV in rural South Africa: a cluster randomised trial. Lancet. 2006 Dec 2;368(9551):1973-83.
Rhodes KV, Rodgers M, Sommers M, Hanlon A, Chittams J, Doyle A, Datner E, Crits-Cristoph P. Brief Motivational Intervention for Intimate Partner Violence and Heavy Drinking in the Emergency Department: A Randomized Clinical Trial. JAMA. 2015;314(5):466-477.
Rothman EF, Wang N. A feasibility test of a brief motivational interview intervention to reduce dating abuse perpetration in a hospital setting. Psychol Violence. 2016 July ; 6(3): 433–441.
Rothman EF, Corso PS. Propensity for intimate partner abuse and workplace productivity: why employers should care. Violence Against Women. 2008 Sep;14(9):1054-64.
Saftlas AF, Harland KK, Wallis AB, Cavanaugh J, Dickey P, Peek-Asa C. Motivational interviewing and intimate partner violence: a randomized trial. Ann Epidemiol. 2014 Feb;24(2):144-50.
Salazar LF, Vivolo-Kantor A, Hardin J, Berkowitz A. A Web-Based Sexual Violence Bystander Intervention for Male College Students: Randomized Controlled Trial. Med Internet Res 2014;16(9):e203.
Sharps P, Alhusen JL, Bullock L, Bhandari S, Ghazarian S, Udo IE, Campbell J. Engaging and Retaining Abused Women in Perinatal Home Visitation Programs. Pediatrics 2013;132:S134–S139.
Stover CS. Fathers for Change for Substance Use and Intimate Partner Violence: Initial Community Pilot. Fam Process 2015 Feb 12. [Epub ahead of print].
Stuart GL, McGeary J, Shorey RC, Knopik VS. Genetics Moderate Alcohol and Intimate Partner Violence Treatment Outcomes in a Randomized Controlled Trial of Hazardous Drinking Men in Batterer Intervention Programs: A Preliminary Investigation. J Consult Clin Psych. 2016, Vol. 84, No. 7, 592–598.
Sullivan PS, White D, Rosenberg ES, Barnes J, Jones J, Dasgupta S, O'Hara B, Scales L, Salazar LF, Wingood G, DiClemente R, Wall KM, Hoff C, Gratzer B, Allen S, Stephenson R. Safety and acceptability of couples HIV testing and counseling for US men who have sex with men: a randomized prevention study. J Int Assoc Provid AIDS Care. 2014 Mar-Apr;13(2):135-44.
Taft CT, Macdonald A, Creech SK, Monson CM, Murphy CM. A Randomized Controlled Clinical Trial of the Strength at Home Men’s Program for Partner Violence in Military Veterans. J Clin Psychiatry 2016; 77(9):1168-1175.
Tiwari AFY, Fong DTY, Wong JTY. Effect of a purpose-built intervention for mental health of mainland Chinese immigrant women who are survivors of intimate partner violence: a randomised controlled trial. Lancet 2015 386(S9).
Tiwari A, Fong DY, Yuen KH, Yuk H, Pang P, Humphreys J, Bullock L. Effect of an advocacy intervention on mental health in Chinese women survivors of intimate partner violence: a randomized controlled trial. JAMA. 2010 Aug 4;304(5):536-43.
Tiwari A, Chan KL, Cheung DS, Fong DY, Yan EC, Tang DH. The differential effects of intimate terrorism and situational couple violence on mental health outcomes among abused Chinese women: a mixed-method study. BMC Public Health. 2015 Mar 31;15:314.
Tollefson DR, Phillips I. A Mind-Body Bridging Treatment Program for Domestic Violence Offenders: Program Overview and Evaluation Results. J Fam Viol (2015) 30:783–794.
Tsai AC, Kakuhikire B, Perkins JM, Vorechovska D, McDonough AQ, Ogburn EL, Downey JM, Rangsberg DR. Measuring personal beliefs and perceived norms about intimate partner violence: Population-based survey experiment in rural Uganda. 2017 PLoS Med 14(5):e1002303.
Van Parys AS, Deschepper E, Michielsen K, Temmerman M, Verstraelen H. Prevalence and evolution of intimate partner violence before and during pregnancy: a cross-sectional study. BMC Pregnancy and Childbirth 2014, 14:294.
Waagman JA, Gray RH, Campbell JC, Thoma M, Ndyanabo A, Ssekasanvu J, Nalugoda F, Kagaayi J, Nakigozi G, Serwadda D, Brahmbhatt H. Effectiveness of an integrated intimate partner violence and HIV prevention intervention in Rakai, Uganda: analysis of an intervention in an existing cluster randomised cohort. Lancet Glob Health. 2015 Jan;3(1):e23-33.
Weir BW, O’Brien K, Bard RS, Casciato CJ, Maher JE, Dent CW, Dougherty JA, Stark MJ. Reducing HIV and Partner Violence Risk Among Women with Criminal Justice System Involvement: A Randomized Controlled Trial of Two Motivational Interviewing-based Interventions. AIDS Behav. 2009;13:509–522.
Wolfe DA, Crooks C, Jaffe P, Chiodo D, Hughes R, Ellis W, Stitt L, Donner A. A school-based program to prevent adolescent dating violence: a cluster randomized trial. Arch Pediatr Adolesc Med. 2009 Aug;163(8):692-9.
Zlotnick C, Capezza NM, Parker D. An interpersonally based intervention for low-income pregnant women with intimate partner violence: a pilot study. Arch Womens Ment Health. 2011 Feb;14(1):55-65.
